# The Role of Semantic Diversity in Word Recognition across Aging and Bilingualism

**DOI:** 10.3389/fpsyg.2016.00703

**Published:** 2016-05-17

**Authors:** Brendan T. Johns, Christine L. Sheppard, Michael N. Jones, Vanessa Taler

**Affiliations:** ^1^Department of Communicative Disorders and Sciences, University at Buffalo, BuffaloNY, USA; ^2^Bruyère Research Institute, OttawaON, Canada; ^3^Department of Psychological and Brain Sciences, Indiana University, BloomingtonIN, USA; ^4^School of Psychology, University of Ottawa, OttawaON, Canada

**Keywords:** semantic richness, cognitive model, bilingualism, memory, word recognition, aging

## Abstract

Frequency effects are pervasive in studies of language, with higher frequency words being recognized faster than lower frequency words. However, the exact nature of frequency effects has recently been questioned, with some studies finding that contextual information provides a better fit to lexical decision and naming data than word frequency (Adelman et al., 2006). Recent work has cemented the importance of these results by demonstrating that a measure of the semantic diversity of the contexts that a word occurs in provides a powerful measure to account for variability in word recognition latency (Johns et al., 2012, 2015; Jones et al., 2012). The goal of the current study is to extend this measure to examine bilingualism and aging, where multiple theories use frequency of occurrence of linguistic constructs as central to accounting for empirical results (Gollan et al., 2008; Ramscar et al., 2014). A lexical decision experiment was conducted with four groups of subjects: younger and older monolinguals and bilinguals. Consistent with past results, a semantic diversity variable accounted for the greatest amount of variance in the latency data. In addition, the pattern of fits of semantic diversity across multiple corpora suggests that bilinguals and older adults are more sensitive to semantic diversity information than younger monolinguals.

## Introduction

Bilingualism is at least as prevalent as monolingualism, with more than 50% of the world’s population estimated to be bilingual or multilingual (Grosjean, 2008). The different experience that bilinguals have with language leads to differences in cognitive functioning between bilinguals and monolinguals, with bilinguals exhibiting lower performance than monolinguals on language-related tasks, but better performance on tasks of executive control (for a review, see Bialystok et al., 2008; Bialystok, 2009; although these advantages are not universal, see Morton and Harper, 2007; Kousaie et al., 2014).

Bilingual disadvantages across language related tasks include poorer naming performance in standardized picture naming tasks (Kohnert et al., 1998; Sheppard et al., 2015), reduced category fluency (Portocarrero et al., 2007), and increased tip-of-the-tongue retrieval failures (Gollan and Acenas, 2004), among others. One influential theory, the frequency lag hypothesis (Gollan et al., 2005, 2008, 2011) proposes that these bilingual disadvantages are due to lower experience with words in one language relative to monolinguals, due to the necessity of using two languages. Lower frequency of a specific word form then leads to slower retrieval times and increased errors in lexical access. Thus, differences between monolinguals and bilinguals reflect the differences in the language environment to which they are exposed, even though monolinguals and bilinguals likely have the same amount of experience with language overall.

Frequency effects are ubiquitous in language processing, with higher frequency being associated with greater speed of processing (e.g., Broadbent, 1967; Forster and Chambers, 1973), and frequency has thus played a central role in models of lexical access (e.g., Morton, 1969, 1979; Norris, 1994, 2006; Goldinger, 1998; Murray and Forster, 2004). Most models assume that each repetition of a word in the language environment increases the strength of that word’s lexical entry, which in turn makes retrieving and processing that word easier. Although different models have different mechanisms to account for the effects of frequency on lexical processing, almost all models incorporate word frequency in some way.

A similar proposal to the frequency lag hypothesis has recently been put forward in the aging literature by Ramscar et al. (2014), who suggest that the slow-down on many psychometric tests that is seen across aging is not due to any systematic decline in the cognitive system, but instead reflects the accumulation of linguistic knowledge across time. As a speaker ages they necessarily have greater levels of experience, which leads to more linguistic knowledge being accumulated, increasing the amount of information that must be processed and thus the time needed to complete a task, due to greater memory search requirements. That is, the aging-related slowing observed in many psychometric tasks simply reflects an increase in the amount of information possessed by the individual, rather than a sign of cognitive decline.

Combined, these two approaches suggest that the amount of experience with language is the central component in accounting for differences in lexical access in both bilingualism and aging (see also Gollan et al., 2008), with the frequency of a word being an important organizational principle. However, the exact nature of frequency effects has recently been questioned on a number of grounds (e.g., Baayen, 2010). In one line of research, Adelman et al. (2006) demonstrated that a measure that estimates a word’s strength in memory by counting the number of contexts in which it occurs (operationalized as the number of document occurrences across a corpus) provides a superior fit to recognition latency over raw frequency.

However, the document count measure of Adelman et al. (2006) ignores an important linguistic information source: the semantic diversity of the contexts that a word occurs in. For example, the word *bank* can occur in the context of a *financial institution* or as a *river bank*, with these discourse topics having different levels of relative occurrence. A document count would count these as the same, but it seems intuitive that this type of semantic diversity should have a role in the lexical system. Even though *bank* is a homograph, this is true of all words to an extent, where there is a natural diversity in the semantic composition of the contexts that a word occurs in.

To more closely examine the role that this diversity plays in language learning, Jones et al. (2012) conducted an artificial language learning experiment that manipulated word frequency and contextual diversity, such that certain words occurred with different sets of words (high semantic diversity), while others repeatedly occurred with the same set (low semantic diversity). While there was no effect of diversity for low-frequency words, high frequency words were retrieved more quickly when they had been learned across multiple diverse contexts, indicating that processing savings only occurred with a change in context. On the basis of these results, and a corpus analysis, Jones et al. (2012) proposed a new model that builds a more accurate measure of a word’s strength in memory based on the degree of semantic redundancy in the set of documents in which a word occurs. Words that occur in more redundant contexts tend to have a lower memory strength than words that occur in more unique semantic contexts. Jones et al. (2012) termed this model the semantic distinctiveness model (SDM). Although we have been using the term semantic diversity to refer to these studies, the exact mechanisms of the model were inspired by work done on distinctiveness effects in memory research (e.g., von Restorff, 1933). Thus, here semantic distinctiveness refers to the mechanism that is used to measure the semantic diversity of a word within a corpus.

The SDM model builds a word’s strength in memory by weighting each new context relative to how much unique information that context provides about the meaning of the word (see below for a full formal description of the model). Across various corpora, this model was able to account for a larger amount of variance to a mega dataset of lexical decision and naming times (the English lexicon project; Balota et al., 2007) over raw word frequency and a document count. Additionally, Johns et al. (2012) demonstrated that this advantage for a semantic diversity count extends to spoken word recognition performance, suggesting that contextual variability is a general property of lexical organization. Johns et al. (2015) recently extended the results of the artificial language experiment of Jones et al. (2012) with natural language materials, further cementing the importance of semantic diversity in natural language processing. Similar studies have explored the importance of semantic diversity across a diverse range of areas, such as in age of acquisition effects (e.g., Hills et al., 2010; Hills, 2012).

However, the importance of semantic diversity has only been demonstrated on tests of young English speakers. Given the central role of differential language experience in both Gollan et al.’s (2008) model of bilingualism and Ramscar et al.’s (2014) account of language processing in aging, it is natural to question what role semantic diversity may play in these different groups. The possibility pursued here is that the bilinguals’ ability and requirement to switch between different languages (Gollan and Ferreira, 2009), leads to a greater ability to discriminate between contexts and in turn use that information in lexical organization to cue which language to use. This increased use of context would lead to an increase in the influence of contextual variability in lexical organization. Additionally, given that bilinguals have an overall lower level of experience with words in a given language (due to the need to split time between two languages; Gollan et al., 2011), it follows that bilinguals may compensate for this lower level of experience by incorporating other useful linguistic information sources, such as contextual information, to a greater degree in lexical organization than monolinguals.

For older people, as Ramscar et al. (2014) point out, there is an increase in the amount of lexical knowledge stored in memory. This would include contextual information, which should also lead to a greater use of this information source in lexical organization.

Thus, it is predicted that both young bilinguals and older monolinguals should show a greater sensitivity to the SD measure. However, given that older bilinguals have both an increased requirement for contextual information (due to the need to language switch) and an increased amount of experience with language (due to aging), it is probable that older bilinguals should show the greatest usage of contextual information in lexical organization, relative to the other subject groups, a prediction that is explicitly tested here.

The goal of the current article is to test the hypothesis that the greater amount of linguistic experience that bilinguals and older adults have received will lead to more sensitivity to contextual information in the lexical access system, based on the models of Gollan et al. (2008, 2011) and Ramscar et al. (2014). We collected lexical decision data from four groups of subjects: younger and older monolinguals and bilinguals. Word frequency, contextual diversity, and semantic diversity measures were derived from a number of different corpora, representing a diverse selection of language. We predicted that younger bilinguals and older monolinguals should have an increase in the fit of the SDM model, as compared with younger monolinguals, given similarities in their amount of lexical experience. Because older bilinguals combine the experiential advantages of multiple languages and increased amount of experience, it is predicted that this group should see the largest advantage for contextual information in lexical organization.

### The Semantic Distinctiveness Model (SDM)

The fundamental operation of the SDM is the use of an expectancy-congruency mechanism when building a word’s semantic representation. Specifically, the encoding strength for a word in a given context is relative to the information overlap between the current environmental context and the representation of a word in memory. This mechanism is very similar in principle to models that adjust attention across learning to dimensions that are more diagnostic (e.g., Kruschke, 1992), which in turn are similar to multiple types of models in learning theory (e.g., Rescorla and Wagner, 1972; Jamieson et al., 2012).

The basic representation is a Word × Document frequency matrix which simply records the documents in which a word occurs. A word’s meaning is represented by the row in the matrix corresponding to that word, a standard approach in computational studies of lexical semantic memory (e.g., Landauer and Dumais, 1997; Griffiths et al., 2007). When a new document is encountered, a new column is added to the matrix. If a word does not occur in the document, it is assigned a value of 0 for that column. If a word does occur in that document, its value for the current context is computed as the sum of the semantic representations of all words that occurred in the document:

$$Context=\overset{n}{\underset{i=1}{\Sigma }}T_{i}\,$$

Where *n* is the number of words in the document and **T_**i**_** is the memory vector of a particular word in the document. The strength with which the word is then encoded into the new column is determined by the similarity of the current context to the word’s semantic representation—the higher the similarity, the less strongly the word is encoded. That is, if the semantic content of the context is redundant with previously stored information, it does not need to be encoded as strongly, as the memory store already contains this information.

Similarity is computed as the vector cosine between the word’s existing memory row and the context. The cosine is passed through an exponential transformation such that high similarity is transformed into low distinctiveness, and low similarity of context is transformed into high distinctiveness. The magnitude of the transformation is controlled by the λ parameter, which is a scaling parameter that determines how much to weight the differences between high and low similarity contexts. This transformed value is the semantic distinctiveness, SD:

$$SD=e^{-\lambda *\cos\left(context,{word}_{i}\right)}\,$$

The *SD* value is then encoded into a word’s row in the new column in the Word × Document memory matrix.

**FIGURE 1:**
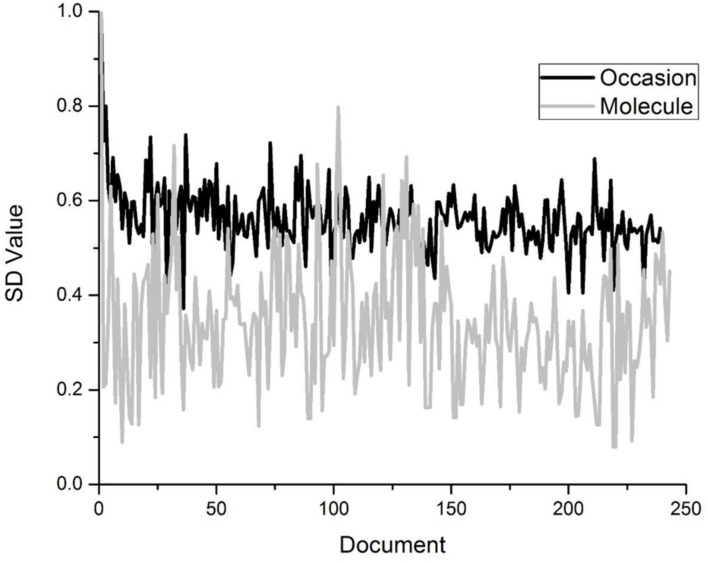
**A visualization of how words differ by their semantic distinctiveness, even when they occur with equal frequency**.

A word’s overall semantic distinctiveness is then simply the sum of the word’s vector elements, i.e., its magnitude. Words that occur in more semantically unique contexts will have a higher magnitude than words that appear in redundant contexts, given equal frequency. An example of this is contained in **Figure 1**, which demonstrates the SD values across the TASA corpus (a standard corpus used in distributional models of semantics; Landauer and Dumais, 1997) for the words *molecule* and *occasion*. Both words appear equally often in the corpus, but the figure demonstrates that the word *occasion* is much more contextually diverse than *molecule*, which tends to occur in more semantically redundant contexts. Across time, this leads to *occasion* having a greater strength in the lexicon. This demonstrates that even though words can have similar numbers of occurrences within language, they can have quite different levels of semantic diversity across their occurrences.

As a first demonstration of our hypothesis that greater linguistic experience leads to greater usage of contextual information, the effect of increasing the contextual resolution of the SDM model was tested on a publicly available set of lexical decision data from younger and older adults. To accomplish this, the increase in the amount of unique variance that the SDM accounts for over both a word frequency and document count variable (measured through percent change in the *R*^2^ value in a multiple regression) was measured as a function of the number of words contained in the model’s lexicon.

In the SDM, as the number of words in the model’s lexicon is increased, the contextual representation that the model forms becomes more refined. Thus, as more words are added into the lexicon, the contextual information that the SDM encodes has a corresponding increase in resolution. The effect of increasing contextual resolution was evaluated using 2,900 item-level lexical decisions times for young and older adults obtained from Balota et al. (1999). Our hypothesis that greater experience with language leads to better contextual learning predicts that older adults should see a larger advantage to increasing the resolution of a contextual representation, as the model’s SD variable becomes more refined. Words were added into the lexicon on the basis of ordered frequency. The SDM model was trained on the TASA corpus (Landauer and Dumais, 1997).

**FIGURE 2:**
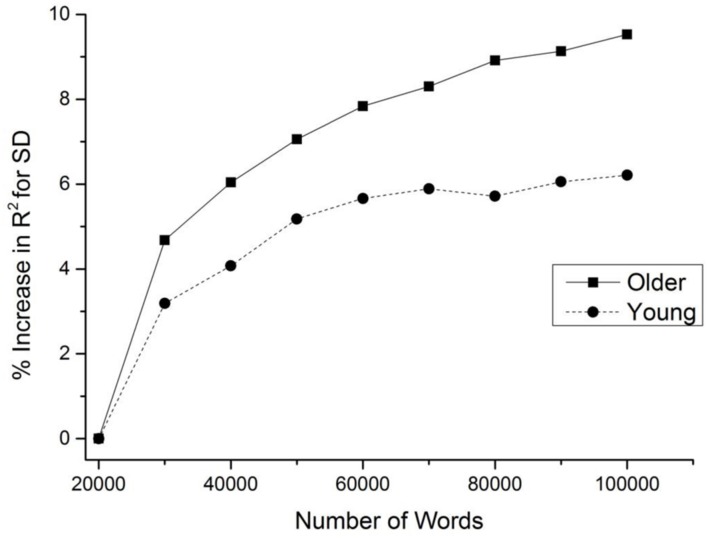
**Increase in the fit of the semantic distinctiveness model (SDM) model to young and older adult lexical decision data as a function of the number of words in the model’s lexicon**.

**Figure 2** displays the results of this demonstration, and shows that both young and older adults experience a benefit as the number of words in the lexicon is increased, at least initially. However, this advantage is larger and hits asymptote at a slower rate for older than younger adults. This result suggests that older adults have a greater ability than young adults to form higher resolution contextual representations, leading to a better ability to harness this information in lexical organization. The finding thus provides initial evidence that an increased experience with language leads to greater usage of contextual information in lexical organization; this hypothesis is tested further here.

## Materials and Methods

### Participants

Four groups of participants were included in this study: monolingual (*n* = 21) and bilingual (*n* = 28) younger adults (aged 18–30) and monolingual (*n* = 16) and bilingual (*n* = 21) adults (aged 65+). All participants had good self-reported health, normal or corrected to normal visual function and no neurological or psychiatric history. All monolingual participants spoke no language other than English, and bilingual participants spoke English and French but no other languages. All bilingual participants acquired a high degree of proficiency in both English and French before age 13 and provided a self-report ranking, on a 5-point Likert scale, of their proficiency in both languages in the area of auditory comprehension, reading, speaking and writing (1 = no ability and 5 = native like ability). Mean self-reported proficiencies for all modalities are provided in **Table 1.** Participants were recruited from the Ottawa–Gatineau, Canada region through advertising and word of mouth. Demographic and neuropsychological data for each group are provided in **Table 2.** Collection of this data was approved by the Research Ethics Board at the Bruyère Research Institute (protocol M16-10-010) and the University of Ottawa (protocol A05-10-27).

**Table 1:** Mean ranking (±standard deviation) for proficiency by modality for younger (*n* = 21) and older (*n* = 28) bilingual participants in English, French, L1, and L2.

		Listening	Reading	Speaking	Writing
Younger adults	English	4.89 ± 0.31	4.82 ± 0.48	4.89 ± 0.31	4.61 ± 0.63
	French	4.89 ± 0.42	4.68 ± 0.55	4.46 ± 0.69	4.34 ± 0.83
	L1	5.0 ± 0.00	4.93 ± 0.26	4.93 ± 0.26	4.79 ± 0.40
	L2	4.79 ± 0.50	4.57 ± 0.63	4.43 ± 0.69	4.16 ± 0.87
Older adults	English	4.90 ± 0.30	4.95 ± 0.22	4.86 ± 0.36	4.81 ± 0.40
	French	4.95 ± 0.22	4.81 ± 0.30	4.86 ± 0.36	4.57 ± 0.51
	L1	4.95 ± 0.22	4.90 ± 0.30	4.90 ± 0.30	4.76 ± 0.44
	L2	4.90 ± 0.30	4.86 ± 0.36	4.81 ± 0.40	4.62 ± 0.50


*Ranking followed a 5-point Likert scale (1 = no ability; 5 = native-like ability).*

**Table 2:** Participants’ demographic, neuropsychological and language characteristics (reported as mean ± standard deviation).

	Younger adults		Older adults		Group comparisons^∗^
				
	Monolingual (*n* = 21)	Bilingual (*n* = 21)	Monolingual (*n* = 16)	Bilingual (*n* = 28)	
Age (years)	21.38 ± 1.12	21.68 ± 2.52	74.06 ± 7.50	70.62 ± 5.70	
Education (years)	15.48 ± 1.08	15.54 ± 1.45	15.06 ± 3.30	16.10 ± 1.27	
Sex (M/F)	7/14	11/17	6/10	14/7	
MoCA (/30)	28.67 ± 1.28	27.93 ± 1.72	27.06 ± 1.88	27.71 ± 1.27	YA > OA
Digit span					
Forward (/16)	10.90 ± 1.61	11.07 ± 2.28	11.00 ± 1.56+	9.81 ± 1.91	NS
Reverse (/14)	6.71 ± 1.98	7.68 ± 2.45	7.80 ± 2.68+	6.81 ± 1.94	NS
WCST (/6)	4.33 ± 1.20	4.58 ± 1.03	3.93 ± 0.96+	3.67 ± 1.11	YA > OA
Stroop					
Word Naming	113.71 ± 15.52	109.19 ± 12.51	94.40 ± 13.07+	98.24 ± 14.82	YA > OA
Color Naming	84.10 ± 13.65	74.63 ± 8.97	63.27 ± 12.90+	58.81 ± 12.20	YA > OA; Mono > Bil
Interference	54.14 ± 11.38	51.89 ± 7.15	34.74 ± 7.4+	36.57 ± 9.07	YA > OA; Mono > Bil
BNT (/60)	53.33 ± 3.28	50.18 ± 5.60	55.87 ± 2.92+	51.71 ± 4.81	NS
Verbal Fluency					
FAS	40.38 ± 14.06	37.04 ± 9.86	43.40 ± 7.43+	40.43 ± 10.42	NS
Animal	26.29 ± 5.32	24.57 ± 5.69	21.47 ± 5.87+	18.81 ± 4.64	YA > OA; trend for Mono > Bil (*p* = 0.07)


*^∗^All comparisons significant at *p* < 0.05; NS, not significant; YA, younger adult; OA, older adult; Mono, monolingual; Bil, bilingual. ^+^Data missing for one participant.*

### Neuropsychological Battery

All participants completed a neuropsychological battery that included the Montreal Cognitive Assessment (Nasreddine et al., 2005); the forward and backward digit span subtests of the Wechsler Adult Intelligence Scale-Third Edition (Wechsler, 1997); the Wisconsin Card Sorting Test (Grant and Berg, 1948); a version of the Stroop test (Stroop, 1935), wherein the number of items produced in 45 s was recorded for each of three conditions (word reading, color naming, and incongruent color naming); the Boston Naming Test (Kaplan et al., 1983); and category (animal) and letter (FAS) verbal fluencies (Benton and Hamsher, 1976). Tasks were completed in English for all participants, including the bilinguals. Scores by participant group are provided in **Table 2.**

### Stimuli

Word stimuli were 300 lexical items in English selected to represent a range of number of features. Norms for the number of features were taken from McRae et al. (2005). Norms for familiarity, concreteness, and imageability were taken from the MRC Psycholinguistic Database (Coltheart, 1981), norms for frequency were taken from the CELEX database (Baayen et al., 1993), and norms for phonological and orthographic neighbourhood density (ND), and bigram frequency by position were taken from the English Lexicon Project database (Balota et al., 2007). These norms were not used in the regression analyses contained below, but were instead used to ensure that the word set used in this study were varied across a number of dimensions. No French-English cognates were included in the stimulus list. Pseudoword stimuli were phonotactically and orthographically legal in English, and were matched to critical stimuli for length, bigram frequency by position, and orthographic ND. Because participants included English–French bilinguals, no pseudoword was a real word in French.

### Procedure

Data were collected using E-Prime software (Version 2.0). Three hundred lexical items (150 words and 150 pseudowords) were presented one at a time in the center of the computer screen, preceded by 18 practice trials (nine words and nine pseudowords). All stimuli were presented in black 24-point Arial font on a white background, and appeared in a different randomized order for each participant. Participants were instructed to decide as quickly and accurately as possible whether or not the item was a real word in English; response was indicated by pressing “a” for real words and “l” for pseudowords. Once the participant made a response, the next stimulus appeared on the screen. Stimuli were set to time out after 2000 msec.

### Corpora and Modeling

Five different corpora were constructed to compare the WF, CD, and SD measures of lexical strength: (1) TASA (Landauer and Dumais, 1997), a standard corpus in the semantic memory modeling literature, consisting of 37,600 paragraphs from texts from textbooks from grades 1 to 12, (2) a 40,000 document Wikipedia corpus, (3) a fiction corpus consisting of 40,000 paragraphs sampled from the works of 15 different authors (spread across 320 books), (4) a non-fiction corpus consisting of 25,000 paragraphs sampled from books from six different discourse topics^1^ (obtained from 200 different books), and (5) a mixed corpus constructed by sampling 25,000 paragraphs from each of the above corpora, meant to represent a more general sampling of language. **Table 3** contains the number of types, number of tokens, and average document size for each of the corpora. The diversity of corpora increase confidence that the results of the model analysis were not due to the construction of a single corpus, but rather hold across a sampling of different language materials. The SDM model was fit independently to each of the above corpora by determining the best λ parameter to 30,000 lexical decision times from the English lexicon project (Balota et al., 2007). There was no trend in the λ parameter across corpora and subject group. WF and CD measures were also attained from these same corpora, in order to do a complete analysis of the different measures.

**Table 3:** Quantitative description of the different corpora used.

Corpora	Number of types	Number of tokens	Average document size	Numbers of documents
TASA	57,800	5,285,933	140.41	37,600
Wikipedia	66,035	7,015,782	175.39	40,000
Fiction	66,632	3,964,482	101.56	40,000
Non-Fiction	60,917	2,860,230	114.41	25,000
Mixed	81,349	13,134,480	131.35	100,000


## Results

### Behavioral Results

**FIGURE 3:**
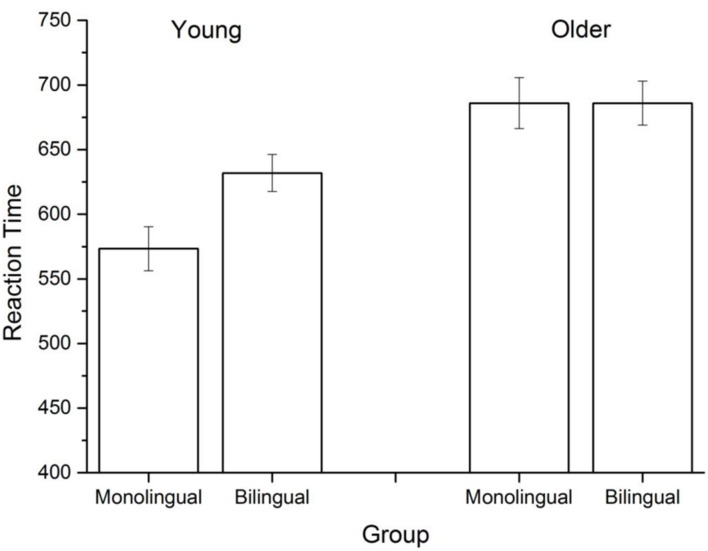
**Mean lexical decision reaction times (RTs) across the different groups**.

To remove outliers, reaction times (RT) were trimmed at 2.5 standard deviations, which across all groups removed 4.87% of all observations. All groups were above 94% accuracy, with no difference being found across groups. **Figure 3** contains the mean lexical decision RTs across the four groups. A 2 (age) × 2 (mono/bilingual) univariate ANOVA revealed a significant main effect of age [*F*(1,85) = 23.605, *p* < 0.001], a marginal effect of bilingualism [*F*(1,85) = 2.911, *p* = 0.09], and a marginal interaction effect [*F*(1,85) = 2.914, *p* = 0.1]. This interaction tendency emerges due to significant differences between young bilinguals and monolinguals, with young monolinguals exhibiting shorter RTs than young bilinguals overall. However, this effect did not emerge in the older participant group. Consistent with previous research, older participants responded significantly more slowly than younger participants. The finding that older adults have higher RTs than younger adults in a lexical decision is well established (e.g., Ratcliff et al., 2004). Longer RTs for younger bilinguals is consistent with previous work comparing bilingual and monolingual lexical access (Ivanova and Costa, 2008) and with the general language processing differences that bilinguals have (Bialystok, 2009). In sum, the behavioral effects replicated standard findings in the literature: younger bilinguals responded more slowly than younger monolinguals, and older participants responded more slowly than younger participants.

### Modeling Results

The first step in the modeling analysis was to determine if the corpora used in the analysis replicate the results of Johns et al. (2012) and Jones et al. (2012), where the SD measure was found to account for more variance in lexical decision times in the English lexicon project (ELP; Balota et al., 2007). The analysis methods employed in this paper simulated those used by Adelman et al. (2006) and Jones et al. (2012). As in these other studies, all WF, CD, and SD values were each transformed to a log scale. The effect of each variable was assessed in a multiple regression analysis where the amount of unique variance over and above the other lexical strength variables was measured through percent change in the *R*^2^ value. **Table 4** contains the amount of unique variance explained by WF, CD, and SD for 30,000 data points from ELP. This table demonstrates the standard finding in this type of analysis: SD accounts for the greatest amount of variance across every corpus, CD occasionally accounts for some additional unique variance, and the effects of WF are largely subsumed by the other variables. This test simply ensures that the word statistics derived from the corpora used here are consistent with past results.

**Table 4:** Unique variance predicted by word frequency, contextual diversity, and semantic distinctiveness for data attained from the English lexicon project.

Corpora	Effect (Δ*R*^2^ in %)		
	
	WF	CD	SD
TASA	0.2^∗∗^	0.657^∗∗^	4.61^∗∗∗^
Wikipedia	0.16^∗∗^	0.53^∗∗∗^	4.43^∗∗∗^
Fiction	0.78^∗∗∗^	1.62^∗∗∗^	5.42^∗∗∗^
Non-fiction	0.0	0.19^∗∗^	2.95^∗∗∗^
Mixed	0.0	0.0	2.98^∗∗∗^


**N* = 30,000, ^∗∗^*p* < 0.01, ^∗∗∗^*p* < 0.001.*

As a first test of the hypothesis that a greater amount of linguistic experience leads to a greater level of sensitivity to contextual information, the amount of unique variance accounted for by WF, CD, and SD for the 2,900 words for younger and older participants from Balota et al. (1999) was assessed. The results of this analysis are displayed in **Table 5**, and validate our hypothesis – across every corpus, the amount of variance explained by SD is greater for older than younger participants. This suggests that the lexical organization of older subjects is more sensitive to the contextual structure of language, leading to a greater success of the SDM model. This larger sample of items will also serve as a validation for our lexical decision experiment with a smaller sample set contained below.

**Table 5:** Unique variance predicted by word frequency, contextual diversity, and semantic distinctiveness for data from Balota et al. (1999) for young and old subjects.

Data set	Corpus	Effect (Δ*R*^2^ in %)		
		
		WF	CD	SD
Young	TASA	0.0	1.21^∗∗∗^	7.74^∗∗∗^
	Wikipedia	0.11	0.395^∗^	7.51^∗∗∗^
	Fiction	0.663^∗∗^	0.0	8.18^∗∗∗^
	Non-fiction	0.183	0.335^∗^	8.73^∗∗∗^
	Mixed	0.279	0.351^∗^	7.62^∗∗∗^
Old	TASA	0.0	3.01^∗∗∗^	12.04^∗∗∗^
	Wikipedia	0.0	1.68^∗∗^	10.11^∗∗∗^
	Fiction	0.253	0.249	11.88^∗∗∗^
	Non-fiction	0.304	0.408^∗^	11.84^∗∗∗^
	Mixed	0.408	117^∗∗^	12.02^∗∗∗^


**N* = 2,900, ^∗^*p* < 0.05, ^∗∗^*p* < 0.01, ^∗∗∗^*p* < 0.001.*

**FIGURE 4:**
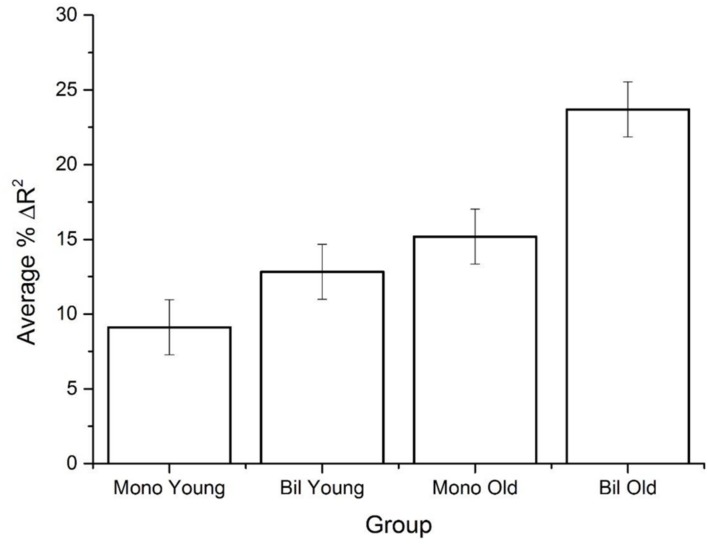
**The average amount of unique variance explained by the SD variable across the five corpora**.

**Table 6** shows the amount of unique variance explained by WF, CD, and SD for the younger and older monolingual and bilingual data collected in our study. As has been found previously, the SD variable accounts for the greatest amount of variance for all subject groups and corpora, while CD accounts for some additional unique variance, and the effects of WF are minimal. Of note is the pattern across the groups in the amount of variance accounted for by the SD variable. The table shows a remarkable consistency across the different corpora, where the amount of variance accounted for follows the same ordinal trend across all corpora: young monolinguals < young bilinguals ≤ older monolinguals < older bilinguals. The average amount of unique variance that the SD variable accounts for across the four groups is shown in **Figure 4**: the differences between groups are quite large, especially for older bilinguals.

**Table 6:** Unique variance predicted by word frequency, contextual diversity, and semantic distinctiveness for young/old monolinguals and bilinguals.

Corpus	Group	Effect (Δ*R*^2^ in %)		
		
		WF	CD	SD
TASA	Mono young	2.11	5.43 *	9.85**
	Bil young	0.0	3.23	11.46***
	Mono old	1.2	5.84	12.15***
	Bil old	0.28	9.03**	21.46***
Wiki	Mono young	0.8	2.01	7.22*
	Bil young	1.98	6.95	14.9**
	Mono old	0	3.33	13.75**
	Bil old	0.47	5.607	18.23**
Fiction	Mono young	0.0	1.273	12.101**
	Bil young	1.73	0.0	17.86***
	Mono old	0.29	1.15	17.01***
	Bil old	0.0	4.61*	31.71***
Non-fiction	Mono young	4.95	8.02*	12.615**
	Bil young	3.514	6.38*	15.974***
	Mono old	11.803**	18.68***	23.27***
	Bil old	5.43	15.48**	30.67***
Mix	Mono young	3.61	5.12*	7.22*
	Bil young	0.3	2.41	9.71**
	Mono old	4.74*	10.02**	13.46***
	Bil old	0.32	7.66*	22.36***


**N* = 150, ^∗^*P* < 0.05, ^∗∗^*P* < 0.01, ^∗∗∗^*P* < 0.001.*

It is possible that this pattern of fits could arise not because the SD variable accounts for more variance in the older bilingual subject’s data, but because all variables offer a poor fit to this group (which would then lead to the SD variable accounting for a proportionally greater amount of the variance). In order to demonstrate that it is not simply the level of fit between the variables and the different subject groups that leads to the differences in variance accounted for by the variables, **Table 7** shows the resulting correlation between the multiple regression model of all three variables for each corpus and data set. This table shows that there is not a large difference in terms of fit for any group. To ensure that the advantage of the SD variable was not due to the difference in the overall fit of the variables across the different groups, the correlation between the values in **Table 7** and the level of unique variance for the SD variable was assessed. The result was non-significant *r*(20) = -0.018, ns, indicating that the amount of variance that the SD variable was accounting for was independent of the overall fit of the different variables to the data.

**Table 7:** Correlation between the multiple regression model that contains word frequency, contextual diversity, and semantic distinctiveness for LDTs across the different groups.

Corpora	*R*			
	
	Mono young	Bil young	Mono old	Bil old
TASA	0.598	0.624	0.651	0.591
Wikipedia	0.443	0.491	0.485	0.452
Fiction	0.56	0.589	0.584	0.607
Non-fiction	0.603	0.559	0.552	0.512
Mixed	0.576	0.575	0.615	0.562
Average	0.556	0.575	0.577	0.542


*All values are significant at the *p* < 0.001 level.*

## General Discussion

The goal of the present study was to examine the role of semantic diversity in word recognition in aging and bilingualism. Lexical decision times across a diverse sample of words were collected from younger and older monolinguals and bilinguals. To determine which type of information source best explained the lexical organization of each groups, a model comparison was conducted across word frequency, contextual diversity (Adelman et al., 2006), and the SDM model (Jones et al., 2012). The SDM provided the closest fit across all subject groups, coherent with past results (Johns et al., 2012; Jones et al., 2012). However, it was the pattern of variance accounted for across the subject groups that proved most interesting; across every corpus, the following trend was observed: young monolinguals < young bilinguals ≤ old monolinguals < old bilinguals. This suggests that these groups’ differential experience with language affects the degree to which contextual variability is used as an organizing cue of the lexicon.

The results of these analyses support our prediction: the differential experience that bilinguals and older adults have with language produces a shift in the type of information used in lexical organization and retrieval. Specifically, there is a difference in the importance of contextual variability with bilingualism and aging. The SDM model still provides the best fit to young monolingual data, identical to past results, but the amount of unique variance explained by the model is greater for young bilinguals and older monolinguals. This is in some ways similar to the results reported in Gollan et al. (2008), where it was found that younger bilinguals resembled older monolinguals more than older bilinguals in performance on a picture naming task. Older bilinguals showed by far the greatest advantage for the SDM model, suggesting that this group uses contextual information to a greater extent than the other groups. These results suggest that as one has more experience with language (and a greater need for contextual information), the ability to discriminate among contexts improves, increasing the use of this information source in language organization, leading to the advantages that are seen here for the SDM model for older and bilingual subjects.

The two theories that motivated the hypothesis that bilinguals and older adults would utilize contextual information to a greater degree are the frequency lag hypothesis of bilingualism (Gollan et al., 2008) and the information accumulation perspective on aging (Ramscar et al., 2014). Both of these theories emphasize the role of differential levels of experience on explaining the deficits that have sometimes been found on language tasks in these groups.

These two theories propose a simple explanation to some seemingly complex issues: the amount of experience that these groups have with different linguistic constructs reflect the difficulty that they have in processing them. In terms of bilingualism, the frequency lag hypothesis posits that bilinguals necessarily have less experience with words in one language relative to monolingual speakers of that language. This difference in experience leads to slower processing times and increased errors in language tasks in bilinguals. In aging, the information accumulation perspective proposes that as we acquire a greater amount of information, it takes longer for the cognitive system to process, due to the greater memory search requirements.

The role of semantic diversity fits in quite naturally with these theories. For bilinguals, contextual information is an important information source for the bilingual lexical access system in order to engage in behaviors such as language switching. That is, contextual information is a necessity when determining which language a person should use, so the higher usage of contextual variability information into the lexical system falls necessarily out of this requirement. The results presented here have supported this view, as it was found that a semantic diversity measure accounts for more variance than other lexical strength measures in lexical decision performance in bilinguals, suggesting that this is a main data source used in organizing the bilingual lexicon.

A similar proposal explains the use of semantic diversity in the aging process. As the demonstration in **Figure 2** shows, as more linguistic information is acquired, there is a concomitant increase in the speaker’s ability to utilize contextual information to organize the lexicon. This suggests that as linguistic experiences accumulate, contextual information becomes more refined, and is used to a greater degree in the lexical system.

Older bilinguals provide an interesting test case of these two proposals. In our analysis, the variability in lexical decision times that the semantic diversity measure accounted for was approximately double for older bilinguals relative to younger bilinguals and older monolinguals. This finding indicates that the increased use of contextual cues in bilingualism and the increased degree of linguistic experience appear to be combinatorial in nature. However, the exact mechanism by which these two processes combine is not entirely clear.

It should be made clear that the SDM is a representational model, and the results here have simply demonstrated that older people and bilinguals use semantic diversity to a greater degree than young monolinguals, consistent with our previously stated hypotheses. This does not provide a mechanistic explanation as to how contextual diversity is used in the lexical access system in older adults and bilinguals. In Johns et al. (2015) it is proposed that the importance of semantic diversity could be due to the use of prediction in the lexical access system (coherent with many other theories of language processing, see Levy, 2008; Altmann and Mirković, 2009; Elman, 2009). The context that one is in provides clues about the words that are likely going to be needed. Words that are low in semantic diversity (so occur in many semantic contexts) would not be as predictive, since they can occur in almost any situation (e.g., *occasion* vs. *molecule* in **Figure 1**). These words should be easier to access since they would not be predictive from context. For older adults, these predictions would become more refined commensurate with experience. For bilinguals, prediction from context would become even more important, as not only do words need to be activated, but the specific language that is required also needs to be activated. Although this conceptualization would allow for the patterns seen in this study to be captured, future research is clearly needed to determine how this is mechanistically possible, but predictive accounts of language provide a promising pathway forward. However, there are a large number of other possible frameworks that could account for these findings.

There are a number of future research questions raised by this work. The most obvious is to determine whether the pattern of advantages for semantic diversity found here also manifest themselves in the artificial language experiment described in Jones et al. (2012) and the natural language experiment described in Johns et al. (2015). If the learning advantages found in tests on young monolinguals are also found in bilinguals and older subjects, and the effects are increased in size in these groups relative to young monolinguals, the overall hypothesis would be supported and additional empirical evidence would bolster the claim of increased use of contextual information in bilingualism and aging.

Another potential path of research suggested by this study comes from the variability in the fits that the different corpora give (see **Table 7**). These corpora represent a highly diverse subset of language, some of which has not been tested before. This led to variability of fits of the different lexical statistics derived from these corpora to the data. Just as the diversity of the local contexts in which words occur is important in lexical processing (as work on semantic diversity demonstrates), it is also probable that the global context in which a word occurs also matters. Different types of corpora may contain different levels of this type of information. For example, a novel contains a narrative, while a non-fiction book tends to be a description of a discourse topic. The way in which these multiple types of texts are processed likely leads to differences in integration of these different language sources into the lexicon. Thus, different behavioral patterns would be observed depending on a person’s past reading experience. How this would manifest in behavioral data is an interesting question for future research.

The role of context in lexical organization has previously been shown to be important in many areas of lexical processing (McDonald and Shillcock, 2001; Adelman et al., 2006; Baayen, 2010; Johns et al., 2012; Jones et al., 2012), but has yet to be extended to the aging and bilingual literatures. The present study constitutes a first exploration of this question. We found that semantic diversity of the contexts in which a word occurs accounted for the greatest amount of variance in lexical decision latencies across all groups, but was found to be especially salient in bilingual and older populations, suggesting that these groups are more sensitive to this information source. This provides support to experiential accounts of bilingualism and aging (Gollan et al., 2008; Ramscar et al., 2014), and suggests that more research should be conducted examining the structure of the linguistic environment and how this manifests in behavioral data across different participant groups.

## Author Contributions

VT and CS designed and conducted the experiments and behavioral analyses. BJ and MJ developed the computational models and simulations. All authors contributed to theoretical development and manuscript composition.

## Conflict of Interest Statement

The authors declare that the research was conducted in the absence of any commercial or financial relationships that could be construed as a potential conflict of interest.

## Footnotes

1 The discourse topics included travel, war, popular science, religion, true crime, and history.
